# A Review of Virtual Coaching Systems in Healthcare: Closing the Loop With Real-Time Feedback

**DOI:** 10.3389/fdgth.2020.567502

**Published:** 2020-09-30

**Authors:** Kostas M. Tsiouris, Vassilios D. Tsakanikas, Dimitrios Gatsios, Dimitrios I. Fotiadis

**Affiliations:** ^1^Biomedical Engineering Laboratory, School of Electrical and Computer Engineering, National Technical University of Athens, Athens, Greece; ^2^Unit of Medical Technology and Intelligent Information Systems, Department of Material Science and Engineering, University of Ioannina, Ioannina, Greece; ^3^Department of Neurology, Medical School, University of Ioannina, Ioannina, Greece; ^4^Department of Biomedical Research, Institute of Molecular Biology and Biotechnology, Foundation for Research and Technology - Hellas, Ioannina, Greece

**Keywords:** virtual coaching, review, coaching systems, automated feedback, closed-loop systems, physical activity monitoring, rehabilitation coaching, behavioral change

## Abstract

This review focuses on virtual coaching systems that were designed to enhance healthcare interventions, combining the available sensing and system-user interaction technologies. In total, more than 1,200 research papers have been retrieved and evaluated for the purposes of this review, which were obtained from three online databases (i.e.,PubMed, Scopus and IEEE Xplore) using an extensive set of search keywords. After applying exclusion criteria, the remaining 41 research papers were used to evaluate the status of virtual coaching systems over the past 10 years and assess current and future trends in this field. The results suggest that in home coaching systems were mainly focused in promoting physical activity and a healthier lifestyle, while a wider range of medical domains was considered in systems that were evaluated in lab environment. In home patient monitoring with IoT devices and sensors was mostly limited to activity trackers, pedometers and heart rate monitoring. Real-time evaluations and personalized patient feedback was also found to be rather lacking in home coaching systems and this is the most alarming find of this analysis. Feasibility studies in controlled environment and an ongoing active research on Horizon 2020 funded projects, show that the future trends in this field are aiming to close the loop with automated patient monitoring, real-time evaluations and more precise interventions.

## Introduction

Coaching systems are on a surge of increasing popularity for both every day and medical applications. The primary driving forces for advancement have been: (a) the enormous growth of access to cheaper smart devices, including smartphones, smartwatches, activity trackers, and in home sensing devices (1), (b) the prevalence of design and development focusing on seamless interconnectivity among them (i.e., Internet of Things, IoT) (2), (c) technology acceptance, as people are becoming increasingly aware and familiar with the added benefits of the functionality that is provided (3), (d) increasing reliable internet access, especially through wireless smartphone connectivity, and (e) the continuous increase in mobile and edge processing power, which raises the possibilities of what can be practically achieved (4). Fitness training and activity monitoring was the killer application that brought massive attention and demand in the field, highlighting the need for more data gathering (i.e., better sensing technologies) and most importantly, the need for advanced data interpretation algorithms and personalized user feedback based on these data (i.e., precise coaching). Machine learning, artificial intelligence and advanced data analytics are the major contributors for making more sense from the sensing data. In the medical and healthcare field, coaching systems have been more relevant for chronic disease patient management and disease modifying behavioral changes, in an attempt to guide and improve intervention outcomes (5).

Virtual coaching is an emerging field in the healthcare domain, which is primarily aiming at improving the personalized user-system interaction. This is crucial for promoting patient engagement and compliance, both of which are necessary for achieving long-term behavioral changes and adaptation of a healthier life-style (6). A virtual interface (e.g., virtual environments, virtual, or augmented reality) can be more effective in creating a more enjoyable experience for the end user, while offering the opportunity to create bounding conditions between virtual objects or human-like avatars. The later can significantly affect the quality of the user-system interaction and adherence to intervention (7). Virtual coaching helps in creating a more immersive experience, which in turn makes patients being more focused in setting and achieving higher goals, instead of following a more lightly-hearted approach.

Virtual coaching can be also most affective in the ends of the age spectrum; children, adolescents, and younger adults on the one end and elders on the other. The first are typically the most aware regarding the latest technological trends and most actively willing to be exposed to new and enticing approaches, including virtual scenarios and gamified coaching solutions. To some extent this is also true for older adults on the other end, but virtual coaching is primarily beneficial for the ease of interpretation it can provide and the enhanced guidance in every step of the intervention. Considering that aging is directly associated with the majority of the neurological disorders that lead to cognitive impairment, coaching strategies for this population group are most often limited in terms of the level of complexity that can be supported (8). The usefulness of the straightforward interactions of a virtual system are also beneficial for young patient populations, who experience equally challenging cognitive and learning difficulties (e.g., Autism spectrum disorders) (9, 10).

Previous review studies in the field of virtual coaching have been conducting focusing on systems that promote self-care (11), physical activity and rehabilitation (12), managing insomnia (13), self-tracking and e-coaching for healthy lifestyles (14), conversational agents and avatars in psychology (15). The main objective of this review is to examine the current status and trends of virtual coaching systems in healthcare, with an emphasis on systems that provide automated patient evaluations and appropriate feedback through virtual environments based on the information being gathered. The primary outcome of this study is to showcase automated coaching systems, use cases of active patient monitoring solutions based on sensing data from IoT devices for advanced feedback, the inclusion of automated real-time evaluations of sensor data (e.g., posture and gait analysis as a subject is moving), and the initiation of appropriate intervention from the virtual coach based on this information (e.g., update instructions, dynamically guide and educate the patient, showcase correct movement when an inappropriate one is detected, etc.). A literature review over the past 10 years is performed to assess recent advancements in the adaptation of such novel technologies for virtual coaching systems that are designed to operate independently in home (i.e., uncontrolled environment), and to investigate future trends based on in lab patient monitoring and coaching technologies that are still under evaluation in feasibility-level studies.

## Methods

### Literature Search Overview

Within this section, the methodology for conducting the scoping review is detailed. More specifically, starting from the research question “Which is, nowadays, the level of validity and maturity of virtual coaching systems and how close are we to fully automated closed-loop systems?,” the methodology proposed in Arksey and O'Malley (16) was followed. The main concept of the present study refers to virtual coaching systems within the medical arena, which collect in an automated way data from the end user (e.g., motion data, speech, gestures, etc.) and, through an inference mechanism, provide feedback related to the scope of each system. Ideally, the coaching systems should be adjustable to each user (i.e., personalized feedback) and be designed to dynamically adapt to its sensing input data, user feedback or environmental conditions. In addition, this review will showcase the use of real-time evaluations and coaching feedback, as this functionality is necessary for more advanced smart virtual coaching systems. Real-time refers to the system's ability to be constantly aware of both the user's condition and its environment, dynamically evaluate his/her actions, behavior, movement along with different environmental factors (i.e., real-time evaluations), and being able to timely respond to such changes as they are being registered with appropriate interventions (i.e., real-time user feedback), to ensure compliance, correct system use as intended, and provide motivation.

The collection of the relative studies was limited to a time range from January 2010 and until April 2020, utilizing three online databases; PubMed, Scopus, and IEEE Xplore. Within this context, a panel of three experts chose the most relevant keywords, which were used for querying the aforementioned databases. The selected keywords were “virtual coach,” “virtual coaching,” “virtual trainer,” “virtual therapist,” “virtual nurse,” “virtual intervention,” “persuasive system,” “virtual reality coaching,” “augmented reality coaching,” “assistive robotics coaching,” “avatar coaching,” “e coach,” and “robotic coaching,” and were used to produce the search terminology for this review, by considering all possible variations. Using these keywords, appropriate search queries were formulated, according to the specifications of each database. It should be noted that the literature review for virtual coaching systems was focused on the medical and clinical domain. In addition, the research papers had to be written in English in order to be included in this review. The type of publication was not considered as a limitation, and all studies that were either published in international journals or conference proceedings were included. The next steps involved the identification of the relevant studies and screening of the search results using a set of exclusion criteria as described below.

### Study Exclusion Criteria

After collecting the literature, three of the authors (i.e., KT, VT and DG) independently screened the titles and the abstracts of all papers, aiming to apply a set of specific exclusion criteria. These criteria involved four main pillars:

- **Domain**: Only coaching systems relating to clinical and medical topics were considered. Thus, coaching systems in the area of e.g., sports were excluded.
- **End users**: Systems should target users who face a morbidity. Thus, any other target group (e.g., medical professionals) were excluded from the next phase.
- **Scope**: The main scope of the presented system should be active virtual coaching. Thus, systems in the domain of telehealth, e-learning, etc. were excluded. Also, systems that did not include any automated inference and feedback mechanism were excluded. The absence of real-time evaluations or real-time coaching feedback was not considered as a factor for excluding a study. Finally, review papers were also excluded.
- **Validity**: Systems with no evaluation/validation study were excluded. In addition, studies reporting findings of coaching systems that were tested on different subject populations than their intended end users were also excluded (e.g., if a post-stroke rehabilitation system was evaluated with healthy individuals).

The review panel met three times, in order to discuss the exclusion criteria and form a consensus on the next steps. During the last meeting, a test among the authors was conducted by randomly selecting ten research papers, from the dataset created in the previous phase. Each author had to exclude all papers of the sample provided that met one or more of the exclusion criteria. All authors pointed out the exact same studies for exclusion. All studies that passed the exclusion criteria were forwarded to the next phase, where a full-text review was conducted, as discussed in the following section.

## Results

### Selected Studies Overview

**Figure 1:**
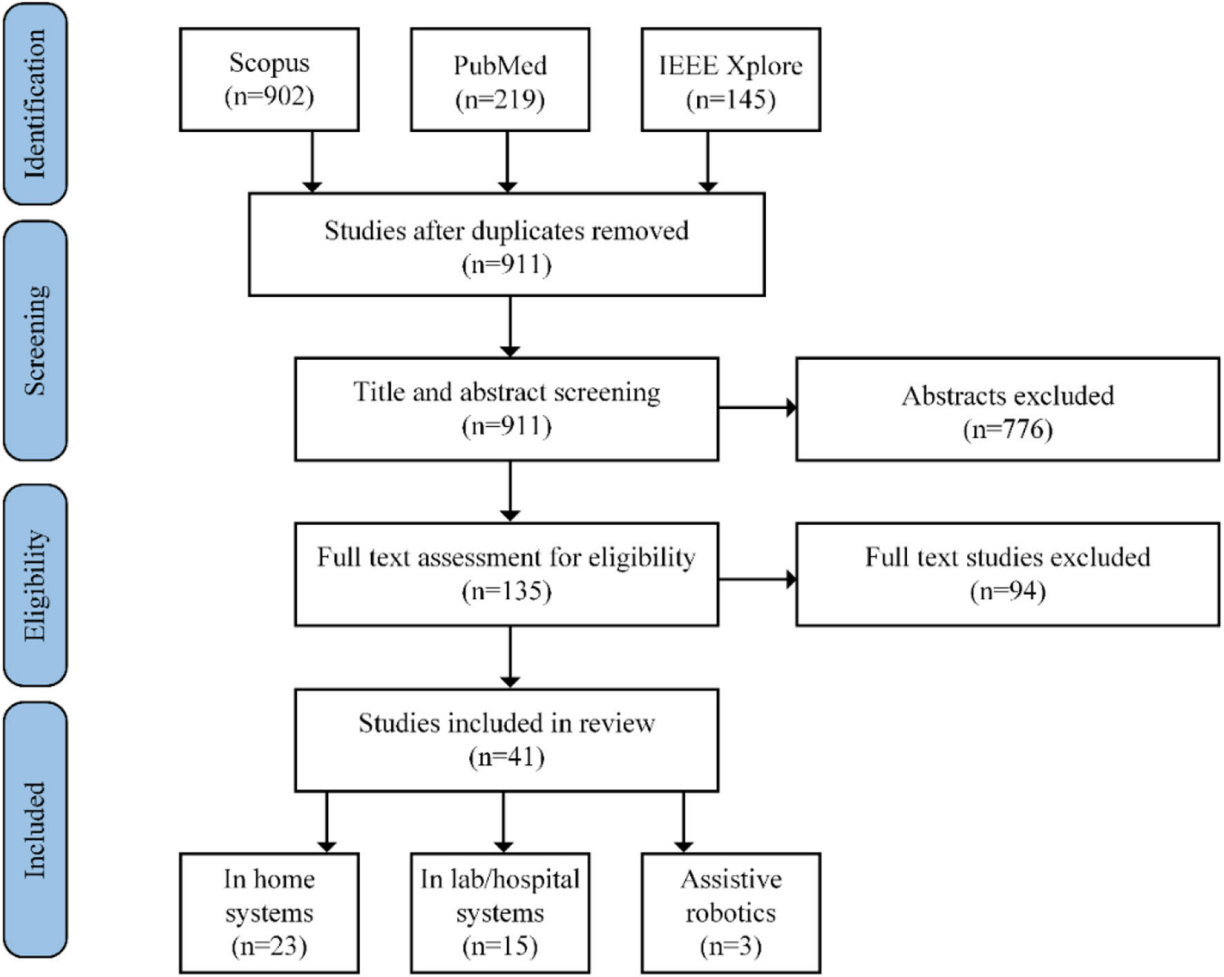
Literature search and screening methodology along with paper selection outcome.

The literature search process from the three databases resulted in a total of 1,266 research papers. From these studies, 71.2% of them were retrieved by the Scopus database, 17.3% from the PubMed database, and 11.5% from the IEEE Xplore database. The steps of the screening methodology are presented in Figure 1, along with the number of studies that were excluded in each step. The first step consisted of removing duplicate records of common studies that were found across the three databases. All unique studies remaining were then considered in the initial title and abstract-based screening following the proposed exclusion criteria (i.e., section Study Exclusion Criteria). At this state, the main exclusion criteria applied involved the domain and the scope criteria. Full text assessment of the remaining papers was then performed. Each one of the three authors involved assessed a total of 50 papers, thus a subset was reviewed more than once to control for inconsistences in the revision process among the panel. At this state, the most relevant exclusion factor was validity, as many studies included only preliminary methods and results, or presented coaching systems without any evaluation results (e.g., studies at pre-pilot state), or systems that were evaluated with subjects different to the intended patient populations.

**Figure 2:**
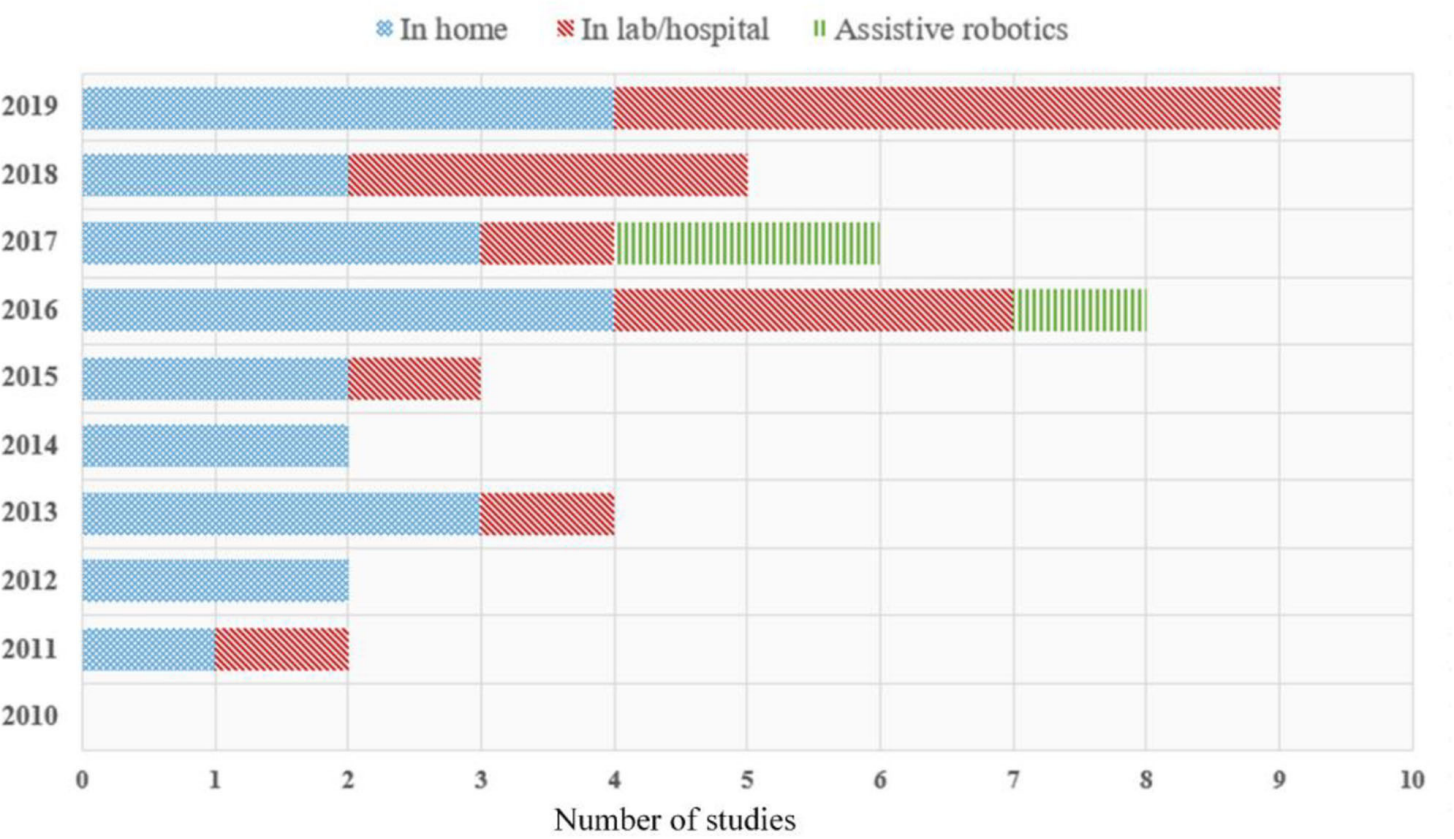
Distribution of selected research papers according to publication year.

**Figure 3:**
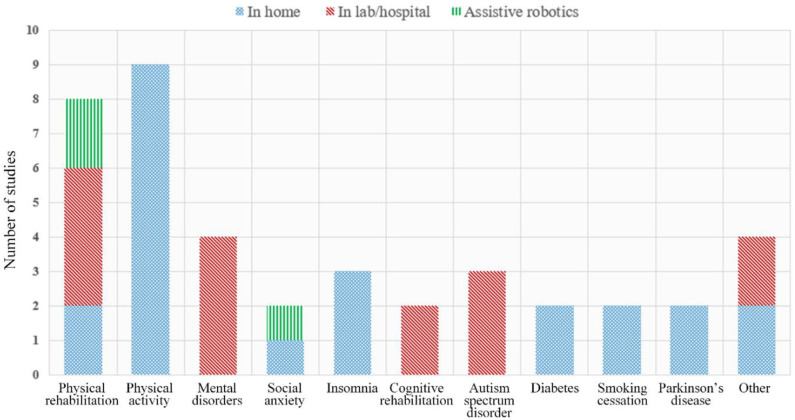
Distribution of selected research papers according to medical domain.

The final subset of the 41 studies selected was then categorized into three classes depending on the targeted evaluation environment and equipment used: (i) home-based systems (56.1%), (ii) lab/hospital-based systems (36.6%), and (iii) systems including assistive robotics (7.3%). Figures 2, 3 present a quantitative schematic representation of the selected papers. More specifically, Figure 2 presents the distribution of papers over time and also separated per class (i.e., home-based, lab/hospital-based, assistive robotics). An increasing trend in the number of studies per year, and therefore virtual coaching systems developed, can be observed within the last decade, and in fact 75.6% of the 41 studies included were published during the second half of the decade. This is a strong indicator that during the next few years, the number of virtual coaching systems will likely further increase. The number of studies per clinical/medical domain is presented in Figure 3, also separated per class.

### In Home Virtual Coaching Systems

Table 1 presents remote virtual coaching systems that were designed and developed to operate at in home environments, as these are prime candidates to showcase the trends and advances in developing state-of-the-art automated models, which are capable of taking advantage of IoT solutions and cloud-edge computing interactions. There are 9 studies presenting coaching systems that focused on promoting and improving the levels of physical activity, with 5 of them focusing on young and middle-aged adults (19, 20, 22, 23, 25), 3 targeting physical activity motivation for the elder population (21, 26, 27), and one study focusing on families with overweight children (24). These applications consisted the typical use case for including continuous active subject monitoring with wearable sensing technology. Systems aiming at reducing the risk of developing type 2 diabetes and weight loss by increasing the level of physical activity in prediabetes subjects and overweight children, respectively, had also implemented continuous monitoring technologies in their intervention programs (24, 33). In most cases, the level of activity was determined by the number of daily steps, while the virtual coaches provided motivational content to help users reach their targeted goals as set during the intervention, in an attempt to keep them engaged in active exercise scheduling, motivate behavioral and lifestyle changes (i.e., especially toward healthier dietary habits) and increase adherence.

**Table 1:** Coaching systems that were designed for and evaluated in home environment.

**Study**	**Healthcare domain**	**UI technologies**	**Coach-user interaction**	**Subject monitoring technologies**	**Continuous monitoring**	**Real-time evaluations and feedback**	**Number of subjects**	**Duration of trial**	**Evaluation outcomes**
Salvi et al. (17)	Physical rehabilitation	Tablet	Motivational virtual coach avatar	ECG, heart rate, respiration rate, accelerometers	-	Heart rate feedback for cardiac rehabilitation	118 adults with myocardial infarction	21 weeks	Improved educational levels but not adherence or exercise habits significantly
Prvu Bettger et al. (18)	Physical rehabilitation	On board screen	Virtual coach avatar to demonstrate and guide activity	3D pose and motion tracking	-	Feedback on exercise quality	287 patients after total knee arthroplasty	12 weeks	Significantly lower health-care costs while providing similar effectiveness
Segerståhl and Oinas-Kukkonen (19)	Physical activity	Wristband display and PC	Exercise level guidance	Heart rate		Heart rate feedback	30 young adults	3 weeks	Feasibility, usability and acceptance of technology study
Watson et al. (20)	Physical activity	PC	Virtual embodied exercise coach	Digital pedometer	Daily number of steps	-	70 overweight adults	12 weeks	Effective in increasing short-term physical activity, long-term effects need further evaluation
Bickmore et al. (21)	Physical activity	Tablet	Virtual embodied conversational exercise coach to motivate walking	Digital pedometer	Daily number of steps	-	263 sedentary older adults	6 months	Effective in increasing short-term physical activity, but problematic long-term maintenance of behavior change
Friederichs et al. (22)	Physical activity	Online program	Motivational interviewing with coach avatar	-	-	-	500 Dutch adults	1 month	If avatar failed to strengthen social relationship with user the impact of the intervention was not enhanced
op den Akker et al. (23)	Physical activity	Smartphone and PC	Feedback by virtual human coach or text	Activity tracker, digital pillbox	Amount of physical activity	-	43 adult office workers	6 weeks	Virtual coach had lower effectiveness compared to text messaging
Gabrielli et al. (24)	Physical activity	Smartphone	Virtual coaching for behavioral change	Activity tracker	Daily number of steps	-	6 families with overweight children	6 weeks	Acceptable solution to support health promotion interventions in primary care
Fadhil et al. (25)	Physical activity	Smartphone	Text-based conversational agent (chatbot)	-	-	-	19 sedentary adults	4 weeks	Preference of using virtual human agent or a combination depends health domain
Brandenburgh et al. (26)	Physical and social activity	Tablet and PC	Virtual coach providing motivational feedback	Accelerometers	Amount of daily activity	-	7 lonely older adults	6 weeks	Reduced loneliness scores
Báez et al. (27)	Physical and social activity	Tablet	Virtual gym environments, personalized avatars, gym coach avatar	Accelerometers, barometric pressure	-	Participation and completion of exercises	34 older adults	10 weeks	High usability and technology acceptance of virtual fitness environment, but poor real-time user interaction
Espie et al. (28)	Insomnia	Online program	Animated personal therapist with personalized advice	-	-	-	164 adults with insomnia	6 weeks	Modest superiority over placebo on daytime sleepiness, improved sleep-wake functioning
Beun et al. (29)	Insomnia	Smartphone	Health coaching dialog system (chatbot)	-	-	-	151 adults with mild insomnia	6 weeks	Significantly more patients reached a meaningful clinical change on Insomnia severity
Lorenz et al. (30)	Insomnia	Online program	Animated conversational coach	-	-	-	56 adults with insomnia	6 weeks	Online CBT had substantial long-term effects on relevant sleep-related outcome parameters
An et al. (31)	Smoking cessation	Online program	Personalized virtual avatar, online peer coach	-	-	-	1698 young adults	12 weeks	Increased smoking abstinence and positive changes in health behaviors
Abdullah et al. (32)	Smoking cessation	Tablet	Virtual conversational agent	-	-	-	6 Veterans	2 weeks	Feasibility study, agent helped in setting a quit date.
Connelly et al. (33)	Diabetes	Online program	Virtual conversational coach	Accelerometers	Level of physical activity	-	31 patients with type 2 Diabetes	6 months	Effective in promoting physical activity, interactive features did not affect activity
Block et al. (34)	Prediabetes	Online program, smartphone	Automated physical activity and eating suggestions and logging	-	-	-	339 adults at risk for developing diabetes	12 months	Improved glycemic control, body weight, BMI, waist circumference, TG/HDL ratio, and diabetes risk
Ellis et al. (35)	Parkinson's disease	Tablet	Virtual embodied conversational exercise coach to motivate walking	Digital pedometer	Daily number of steps	-	20 patients with PD	1 month	High retention, satisfaction and adherence to daily walking, significant improvement in mobility
van der Kolk et al. (36)	Parkinson's disease	PC	Virtual cycling targeting heart rate zone	Static bike with heart rate	-	Heart rate feedback	130 adults with mild PD	6 months	Aerobic exercise at home is feasible and reduced off-state motor signs
Andrade et al. (37)	Overactive bladder	Online program	Virtual human avatars and self-avatar	-	-	-	41 adult women	12 weeks	Improved QoL and overactive bladder symptoms
Hartanto et al. (38)	Social anxiety disorder	Head mounted display	Virtual health agent, dialogues with virtual characters	Head tracker, heart rate, microphone	-	Natural speech recognition, heart rate feedback	5 adults with social phobia	10 sessions	Anxiety was evoked and over time gradually decreased due to exposure therapy, but serious technical problems occurred
Wu et al. (39)	Powered wheelchair seating	Smartphone (mobile app)	Virtual seating coach promoting powered seating functions	Accelerometers	Seating angle monitoring	Reposition adjustment	5 powered wheelchair users	5 days	Feasibility study, accelerometers were unstable for seating angle while moving

*UI, User Interface; Subject monitoring technologies: Type of sensing data/devices used, Continuous monitoring: Always-on tracking features, Real-time evaluations and feedback: Dynamic user evaluations using sensing data and provision of appropriate interventions*.

Insomnia was found to be another common domain for healthcare coaching systems. All interventions relied on conversational systems, delivering personalized advice either through animated coaching avatars (28, 30), or through automated text dialogs (i.e., chatbots) (29). None of these coaching systems included active sleep monitoring and analysis, but relied on self-reported sleep diaries and user information. Availability of sensor-based sleep tracking technologies however is on the rise as it is currently supported by the majority of commercial smart watch manufacturers (e.g., Garmin, Fitbit, Withings, Samsung, Polar, etc.), and such systems could find their way in similar studies in the future, as sleep tracking algorithms continue to mature. The same means of user-coach interaction was used for the two systems that were developed to assist with smoking cessation in Veterans (32) and young smoking adults (31). A similar system based on interactions with virtual human avatars was also developed to develop a self-management program that could assist older women with overactive bladder symptoms, improving quality of life and perceived symptoms severity (37). The primary focus of these systems was to raise awareness and promote behavioral changes for a more healthy lifestyle overall, a concept that led to holistic approaches with conversational agents that targeted the promotion of physical activity, healthy diet and effective stress coping as in the study of (25).

Real-time feedback on the other hand was included in only a few studies. Baez et al., created a virtual gym environment to address both aspects of physical training and social user interactions between older adults, and used wearable sensors to measure adherence by tracking exercise completion (27). Rehabilitation training consisted another medical domain where real-time feedback was provided to the user, for exercise quality evaluation through tracking posture and body movement during home-based physiotherapy programs for patients after total knee arthroplasty (18). More common was the use of heart rate monitoring and cardiac-control during exercise. Coaching systems that were guiding the level of activity based on heart rate monitoring for improved rehabilitation were developed and tested for patients with myocardial infarction (17), and patients diagnosed with Parkinson's disease (36). Focusing on younger adults, Segerståhl and Kukkonen, developed a system that could be used to guide exercise execution and keep the users within a certain cardiac level (i.e., training intensity level), using a wearable heart rate monitoring device (21). A wearable heart rate sensor was also implemented in another system that targeted virtual interventions for subjects with social anxiety disorders using virtual health agent and characters to simulate stressful immersive environments, where patients could train to improve their social behavior skills (38). Finally, in a more specialized medical application, Wu et al. developed a prototype coaching system that could assist powered wheelchair users to take full advantage of the provided powered seating functions (e.g., tilt in space, backrest recline, leg rest elevation), following clinically recommended adjusting protocols to maintain seating stability and prevent prolonged-seating complications (39).

### Coaching Systems for Hospital/Lab Environment

This section includes 15 studies showcasing virtual coaching systems that were designed and evaluated in controlled hospital or lab environments, or virtual coaching systems that were designed for in home environment, but were evaluated in hospital/lab settings. As it shown in Table 2, 11 of these studies consisted of preliminary system evaluations focusing on feasibility and technology acceptance, performing single supervised experimental sessions in small patient populations. The primary focus of the virtual coaching systems that were tested was physical rehabilitation and psychological training for subjects with cognitive impairment, phobias, or mental disorders. Since the experiments were conducted under medical supervision, the clinical use cases extended to patients with more severe physical, mental or cognitive deficits, who were commonly excluded from studies and systems targeting in home evaluations, as the intervention could lead to uncontrolled and hard to predict adverse events. As expected, technologies for continuous subject monitoring could not be properly evaluated under these experimental conditions and, thus, none of these studies reported such measurements. On the other hand, real-time evaluations were extensively integrated in the proposed systems and tested in these studies, with “live” user feedback functionality being the key differentiating factor compared to the in home systems presented in the previous section.

**Table 2:** Coaching systems that were evaluated in controlled laboratory or clinical environment under direct supervision.

**Study**	**Healthcare domain**	**UI technologies**	**Coach-user interaction**	**Subject monitoring technologies**	**Continuous monitoring**	**Real-time evaluations and feedback**	**Number of subjects**	**Duration of trial**	**Evaluation outcomes**
Pirovano et al. (40)	Physical rehabilitation	TV screen	Gamification of training with virtual therapist	Kinect, Wii Balance Board, Tymo Therapy Plate	-	Motion capture and body balance	7 older adults with multi-morbidities	1 session	Feasibility and usability study
Michel et al. (41)	Physical rehabilitation	TV screen	Gamification training with motivational messages from animal virtual coach	Kinect	-	Hand movements	20 children with Cerebral Palsy	4 sessions	Usability and acceptance of technology, game was enjoyable
Lupu et al. (42)	Physical rehabilitation	VR headset, PC monitor	VR avatar of a physiotherapist	Electrical stimulator in arms, EOG, EEG	-	Eye tracking (EOG), motor imagery hand movements	7 adults with post stroke central neuromotor syndrome	3 sessions	Feasibility and technology acceptance study
Pham et al. (43)	Physical rehabilitation	TV screen	Jintronix games and therapy modules	Kinect	-	Motion capture	20 hospitalized adults with burn injuries	1 session	Feasibility study, demonstrated good acceptability and safety in hospital environment
Bell and Weinstein (44)	Mental disorders	PC monitor	Virtual conversational agent providing feedback on interview responses	Headset with microphone	-	Speech recognition	10 subjects with psychiatric disabilities	1 session	Strongly positive response to simulated job interview skill training
Cameron et al. (45)	Mental disorders	PC monitor	Chatbot providing self-assessment and tips for stress, anxiety, depression, sleep, and self esteem	-	-	-	7 employees from a mental health social enterprise	1 session	Enjoyable, easy to use and consistent, but poor error management and intelligence
Fok et al. (46)	Cognitive rehabilitation	PC monitor	Audible and visual cues in virtual environment of a kitchen	Data gloves	-	Position and tactile information of hands	2 post-stroke patients with cognitive impairment	1 session	Observing therapists endorsed the system, but larger population study and improvements needed
Wirzberger et al. (47)	Cognitive rehabilitation	TV screen	Dialogue-based memory training with virtual agent	Microphone	-	Speech recognition (experimenter)	62 older adults	1 session	Training performance correlated with recall, longer system response times showed performance benefits
Freeman et al. (48)	Psychological therapy	VR headset	Avatar coach in VR environment	Microphone, head and hand VR trackers	-	Speech recognition, hand movements	100 adults with fear of heights	4 weeks	VR can increase treatment provision for mental health disorders
Liu et al. (49) Vahabzadeh et al. (50)	Autism spectrum disorder	AR headset	Interaction in augmented reality environment	Smart glasses	-	Gaze attention, head movement	10 children with ASD and their caregivers	1 session	Initial evidence in reducing hyperactivity, inattention and impulsivity in children with ASD
Rosenfield et al. (51)	Autism spectrum disorder	VR headset	Social training in VR environment	VR tracker, microphone	-	Head movement, speech recognition	2 children with ASD	1 session	Feasibility and technology acceptance study
Miloff et al. (52)	Exposure therapy	VR headset	Virtual therapist delivered voiceover psychoeducation and supportive feedback	Smartphone IMU sensors	-	Head movement	100 adults with spider phobia	1 session (52 weeks follow up)	VR therapy reduced short-term symptoms, no long-term advantage over *in-vivo* therapy
Kang et al. (53)	Virtual nurse	PC monitor	Virtual nurse offering healthcare and lifestyle advices	Microphone	-	Speech recognition	26 older adults	1 session	Virtual nurses based on adaptive persuasion models resulted in higher social presence and lower frustration
Shamekhi et al. (54)	Self-care management	PC monitor	Conversational agent as virtual coach	-	-	-	9 adults with spinal cord injury	1 session	Feasibility and technology acceptance study

*UI, User Interface; Subject monitoring technologies: Type of sensing data/devices used, Continuous monitoring: Always-on tracking features, Real-time evaluations and feedback: Dynamic user evaluations using sensing data and provision of appropriate interventions*.

Virtual reality (VR) wearable headsets were introduced into different medical domains, along with more advanced movement tracking and speech recognition systems. Freeman et al. used immersive VR environments to enhance psychological therapy interventions for adults with height phobias by simulating different scenarios of high altitude (48), while subjects with spider phobia were also introduced to a VR system that provided exposure therapy and educational material to help them cope with their fear (52). Systems offering social training for children with Autism Spectrum Disorders (ASD) (51), and physical rehabilitation for post-stroke patients (42) have also been evaluated, showing positive outcomes of the feasibility and acceptability of the immersive VR technology in medical coaching applications. Augmented reality (AR) devices in the form of smart glasses were also tested with children suffering from ASD, as a means to assist them in directing their attention toward a human companion during conversation scenarios, and promote social activities (49, 50).

As it is shown in Table 2, the controlled environment and the supervised experiments allowed researchers to evaluate more complex sensing and monitoring technologies. Virtual coaching and gamification of physical rehabilitation training with advanced movement tracking and real-time head and body movement evaluations were implemented using commercially available devices, including Microsoft's Kinect, Nintendo's Wii platform and its peripherals (40, 41, 43). In addition, in the cases where VR was also part of the intervention, head, and hand movement tracking was also performed using the included proprietary trackers of the commercial VR systems, or the integrated IMU sensor if a smartphone attached to a head-mounted device was used instead. Eye tracking through commercial smart glasses was also implemented in AR systems targeting children with ASD, in order to train them to direct their attention toward a fellow conversational companion and engage more in social activities (49, 50).

Despite being in a more laboratory state of technology readiness, custom tracking solutions were less common and only a couple of studies invested in alternative solutions. In a proof of concept study targeting cognitive rehabilitation, a sensing glove was utilized to test feasibility of a more realistic hand and finger movement tracking system with tactile feedback, while performing an intervention focusing on virtually simulated everyday tasks (e.g., meal preparation), showing mixed results in post-stroke patients (46). In another system for post-stroke rehabilitation developed by Lupu et al., biosignal analysis was used for eye movement tracking and motor imagery training, using electrophysiological data captured while recording patient's electrooculogram (EOG) and electroencephalogram (EEG) signals (42). As it shown by their inclusion in a single study, the use of such technologies for medical applications is currently in its infancy state, as the acquisition of biosignals can only be implemented under direct medical supervision and assistance.

Systems supporting natural speech recognition and text semantics analysis in the form of automated conversational agents either through virtual avatars of chatbots, respectively, were also found to gain research attention for coaching purposes. They were primarily used in systems targeting patients with mental and cognitive disorders, offering healthcare and motivational coaching, in order to help them overcome phobias, mental health care, and train in social activities (44, 45, 47, 53). Speech recognition was commonly used to complement systems with immersive VR, in order to provide an enhanced user-system interaction experience (48, 51).

### Assistive Robotics in Coaching

This section describes the use of external assistive technologies and robotic-based interventions as part of the coaching process. As expected, these systems were evaluated under supervised conditions in lab environment, but are distinguished by the studies reported in section Coaching Systems for Hospital/Lab Environment above, since the added robotic devices enhance the realism of the interventions, especially when it is complemented with automated audio feedback and speech recognition capabilities. Robotic rehabilitation systems could enhance the sensing input for the patients, providing force and haptic feedback, while interactions with actual robotic coaches and older adults that were used in socially assistive robotic systems, were found to be useful in improving social, physical, and cognitive training programs. The later was shown to be very effective in a recent study, where a commercial robot (i.e., NAO robot) was used as a coach to assist and provoke social interactions in older adults with and without cognitive impairment (55). The robotic coach was designed to provide one-on-one coach-user interaction, as well as multi-user and coach interactions, with individualized activity management and dynamically adaptive behavior for long-term engagement and was very received during its initial testing.

Then, there were also two feasibility evaluation studies that focused on robotic physical rehabilitation. Despite targeting different age population groups and medical conditions, both studies resulted in similar findings; showcasing the proposed coaching systems as useful training tools to complement the existing approaches in rehabilitation therapy. Chiang et al., used force feedback from two haptic devices to enhance real-time user-system interactions in non-immersive VR environments, training children and adolescents with upper limb disabilities to improve performance in activities of daily living, showing high level of satisfaction when using virtual rehabilitation systems (56). An arm exoskeleton that provided gravity-compensation in arm movements and dynamically adapted feedback in non-immersive VR environment was also tested for personalized rehabilitation in post-stroke patients with severely affected motor functionality (57). A closed-loop system that facilitated unsupervised motor learning providing adjustable level of supported depending on each patient's motor potential, and continuous visual feedback of a virtual arm was proposed to complement the perception of natural movement and improve the quality and range of the functional restoration.

## Discussion

As Figure 3 suggests, 17 studies of the total of 41 included in this review of virtual coaching systems dealt with either physical rehabilitation or physical activity and motivation of healthier lifestyle choices. This was also found to be the most prominent field for using sensing devices as 15 out of the 17 systems included patient monitoring, especially with the capacity to capture level of exercise with activity trackers, accelerometers, and pedometers, and relevant body movement using depth cameras for precise posture and skeleton tracking, or even haptic and exoskeleton devices. Systems for motivation of physical activity could also be more easily implemented in larger patient populations as they did not engage specific and detailed real-time evaluations and relied mostly on user's self-reported input. Coaching systems for mental disorders and cognitive rehabilitation were also found to gain research attention in the past few years, but, as shown in Table 2, their applications were limited only within supervised testing conditions and have not yet been transported to independent, in home use scenarios. In addition, another notable remark refers to the under-representation of clinical conditions, like Parkinson's disease, cardiovascular diseases, ASD, diabetes, and orthopedics for which technology-aided virtual coaching systems should have a substantial impact to quality of life and everyday activities.

Integration of new technologies for in home coaching systems was also found to be rather limited, which is an alarming outcome for the field, overall. For example, immersive VR and AR interaction solutions were not implemented in systems that targeted in home use cases, and were rather only found in controlled lab environment. This is an indicator, however, that these technologies are currently being meticulously tested, and mass adoption in the future is to be expected. Especially as they continue to improve in terms of supported functionality, tracking accuracy and lower costs. The same could be applied to assistive robotics, as positive results from the studies reported in Table 3 suggest that the robotic-based training has great potential to be used as a way to enhance existing interventions in occupational therapy. Although wide spread adoption is more challenging than VR and AR technologies, there is already evidence that the novelty factor that accompanies assistive robotic interventions, can be very beneficial in specific medical conditions such as stroke rehabilitation (58).

**Table 3:** Coaching systems with assistive robotic functionality.

**Study**	**Healthcare domain**	**Coach-user interaction**	**Subject monitoring technologies**	**Continuous monitoring**	**Real-time evaluations and feedback**	**Number of subjects**	**Duration of trial**	**Evaluation outcomes**
Grimm et al. (57)	Physical rehabilitation	Training with adaptation of task difficulty in virtual environments	Arm exoskeleton (7 degree-of-freedom)	-	Hand movement tracking in interactive games	5 post-stroke patients with upper extremity motor deficits	4 weeks	Gravity-compensation and progressively challenging motor learning can facilitate functional restoration
Chiang et al. (56)	Physical rehabilitation	Interactive user interface with force feedback in virtual environments	Haptic devices (6 degrees of freedom inputs)	-	Interactions in virtual environments	20 students with upper limb dis-abilities	2 months	Useful training tool to complement conventional rehabilitation approaches
Fan et al. (55)	Socially assistive robotics	Interaction with robotic coach	NAO robot, Kinect, EEG, galvanic skin response	-	Gesture and speech recognition, gaze estimation	25 older adults (some with cognitive decline)	1 session	Good system functionality and positive perception of the robotic intervention

*Subject monitoring technologies: Type of sensing data/devices used, Continuous monitoring: Always-on tracking features, Real-time evaluations and feedback: Dynamic user evaluations using sensing data and provision of appropriate interventions*.

Subject monitoring and sensing technologies being used in coaching systems seem to be highly dependent on technology readiness levels of the various IoT devices. The level of physical activity as estimated through step tracking is found to be the most common means, which can be explained considering that it is nowadays a mature technology (i.e., in terms of both hardware and software), with increased availability and reduced associated costs, as competing commercial wearable devices pushed advancements in this field. Microsoft's Kinect was another landmark device that enabled advanced functionality in coaching systems, as it provided unobtrusive monitoring of whole body movement and more precise user-system interaction with upper extremities. Despite being discontinued at consumer level, its practicality allowed even non-highly tech educated people to familiarize with this technology and has inspired researchers to develop medical devices and systems that integrate similar functionality, especially in rehabilitation interventions (18, 59). Heart rate monitoring is another field where IoT devices have reached a level of functional robustness and monitoring accuracy that justified their widespread use for clinical use cases in coaching systems (Table 1). A similar transition in monitoring technology is currently being materialized following the advancements in smartwatches, which should be shown in more precise physical activity and sleep monitoring and enhanced evaluation feedback. This is the first link that will enable the development of personalized and precise monitoring systems that can seamlessly provide continuous patient tracking in their everyday life environment and living conditions.

The second important link in the chain of more advanced coaching systems is the feedback that they can provide to the user. As the distinction of in home and in lab systems in the results section revealed, the transition from offline and semi-real evaluation of user behavior to real-time feedback for in home environment is currently in its early stages (Table 1), despite the widespread use cases of real-time evaluation functionality under controlled, supervised conditions (Table 2). The findings in Table 2 however, are indicative of the future trends in the evaluation feedback of coaching systems, and the necessity to close the loop with automated and more precise real-time evaluations. This would ultimately allow the available clinical personnel to effectively supervise larger patient populations in their medical domain of expertise, and increase access to better healthcare services in rural areas.

The increased practical functionality that the improved monitoring technologies offer (section Coaching Systems for Hospital/Lab Environment), could define a new generation of coaching systems that are able to revolutionize patient perspective, as “smart” systems become progressively aware of user's environment, activities and behavior, improving the quality of user-system interactions and eventually adherence to the intervention protocols. A closed-loop coaching system that can dynamical adjust to each patient's needs, evaluate their behavior and actions in real-time, and intervene accordingly offers a holistic approach in motor and cognitive training and rehabilitation address. This will set a crucial milestone in coaching systems enabling the continuity of healthcare between subsequent visits with medical professionals.

The lack of holistic coaching systems was also acknowledged by the European Commission, which led to several recent projects responding to the H2020 call SC1-PM-15-2017—Personalized coaching for well-being and care of people as they age. The call tried to address the deployment of radically new solutions for personalized virtual coaching systems, building upon intelligent ICT environments, access to relevant physiological, and behavioral data and new forms of accessible interaction based on tangible user interaction concepts. Currently, these projects are evaluating the different approaches with the targeted aging population:

HOLOBALANCE^1^ introduces a new personalized platform for virtual coaching, motivation, and empowerment of older citizens with balance disorders. The coaching part is realized with a holographic surrogate physiotherapist and augmented reality games, along with easy to use wearable sensors in an interoperable platform design (60).
Council of Coaches^2^ introduces a radically new virtual coaching concept based on multiple autonomous, embodied virtual coaches, which form together a personal council addressing the needs of older adults in an integrated manner (61).
WellCo^3^ provides an affective-aware coach that interacts through speech with the user in order to act as a virtually interface among the user and the platform managing the flow of all interactions and empower users in their behavior change process through simulation activities tailored to their current mood (62).
CAPTAIN^4^ proposes a transparent technology designed to turn the home of the older adult into a ubiquitous assistant, based on a projected augmented reality through use of micro-projectors, contextualized information and instructions on top of the real environment (63).
vCare^4^ embeds clinical profiles and the pathways to drive the behavior of the virtual coach at home and provide well-elaborated services for tele-rehabilitation in neurology and cardiology (64).
SAAM^5^ supports the aging population living at home, with a novel and practical emphasis on ambient sensing and learning of user needs and preferences, and effective coaching by leveraging the user's social support networks (65).
EMPATHIC^6^ proposes multimodal face analytics, adaptive spoken dialogue systems and natural language interfaces along with non-intrusive technologies to extract physiological markers of emotional states in real-time, in order to support dependent aging persons and their caregivers (66).
NESTORE^7^ leverages on novel ICT technologies for unobtrusive monitoring system, including wearable and environmental sensors, intelligent Decision Support System to provide personalized goals toward wellbeing, and active coaching as conversational agents, embodied in a physical companion to establish affective communication and engage user in personalized coaching activities (67).

Finally, it should be noted that besides the large amount of studies that has been considered during the literature search, it is still very likely that some relevant research papers have not been discovered or missed. From the studies that have been included however, it is clear that the current and near future research trends in virtual coaching systems are pushing to close the loop with advanced patient monitoring, improved virtual user-system interactions and most critically, support for automated feedback and dynamically adjusted precise interventions, based on personalized evaluations from the sensing data.

## Conclusion

The systematic evaluation of the literature within this review has shown that virtual coaching systems are gaining momentum, as new monitoring and system-user interaction technologies are being evaluated and their medical applications are expanding in more complex diseases. Technology integration is highly dependent on the current status and supported functionality of IoT sensing devices, with novel patient monitoring and intriguing virtual interfaces becoming increasingly popular in coaching systems, especially in controlled and supervised environments. Transition to fully automated in home coaching systems, however, is proving to be in its early stages and many obstacles still remain before holistic coaching solutions with real-time evaluations and personalized feedback can become widely available for unsupervised in home use. The added value of such systems in terms of effectiveness, technology acceptance, and reduced healthcare costs has been well documented though and proven in proof of concept studies over the past few years. The current research projects that were funded to address these issues are on track to set the foundations for a new generation of coaching systems.

## Author Contributions

KT, VT, and DG performed the screening process of the research papers. DF performed a final revision of the final manuscript. All authors contributed equally in the preparation of the manuscript, the formulation of the literature search methodology, the exclusion criteria used for this review, revision of the article, and approved the submitted version.

## Conflict of Interest

The authors declare that the research was conducted in the absence of any commercial or financial relationships that could be construed as a potential conflict of interest.

## Footnotes

^1^ https://holobalance.eu/

^2^ https://council-of-coaches.eu/

^3^ http://wellco-project.eu/

^4^ https://www.captain-eu.org/

^4^ https://vcare-project.eu/

^5^ https://www.saam2020.eu/

^6^ http://www.empathic-project.eu/

^7^ https://nestore-coach.eu/home
